# Apropos: factors impacting on progress towards elimination of transmission of schistosomiasis japonica in China

**DOI:** 10.1186/1756-3305-7-408

**Published:** 2014-08-29

**Authors:** Wei Wang, Jian-Rong Dai, You-Sheng Liang

**Affiliations:** Jiangsu Institute of Parasitic Diseases, 117 Yangxiang, Meiyuan, Wuxi City, Jiangsu Province 214064 People’s Republic of China; Key Laboratory on Technology for Parasitic Disease Prevention and Control, National Health and Family Planning Commission, 117 Yangxiang, Meiyuan, Wuxi City, Jiangsu Province 214064 People’s Republic of China; Jiangsu Provincial Key Laboratory of Molecular Biology of Parasites, 117 Yangxiang, Meiyuan, Wuxi City, Jiangsu Province 214064 People’s Republic of China

**Keywords:** Schistosomiasis, Elimination, China

## Abstract

Currently, China is moving towards the elimination of schistosomiasis japonica. In a previous review, the factors affecting the progress towards the elimination of transmission of schistosomiasis in China have been summarized. Nevertheless, some factors were neglected. Hereby, we describe four other factors which may threaten the achievement of the goal of schistosomiasis elimination in China.

## To the Editor

After nearly 60 years of effort, China has achieved great success in the control of schistosomiasis japonica, and currently, the prevalence and intensity of *Schistosoma japonicum* infection have been reduced to an extremely low level [1, 2]. The elimination of this neglected tropical parasitic disease has been, therefore, put on the governmental agenda [3]. In a previous review, Zhou and colleagues summarized the factors affecting the progress towards the elimination of the transmission of schistosomiasis japonica in China [4]. These factors, in our opinion, may indeed have potential impact on schistosomiasis elimination, and should be taken into account during the development of the strategy targeting the elimination of the disease in China. However, some other factors were neglected. Hereby, we describe four other factors that may threaten schistosomiasis elimination in China, including political and financial support, climate change, praziquantel resistance, and potential transmission of African schistosomiasis.

The experiences and lessons from the past 5 decades of schistosomiasis control in China have shown that political will and financial support are critically important to the effective control of the disease [5]. At the first stage (from mid-1950s to early 1980s) of the progress of schistosomiasis control in China, there was strong political will among the leaders of the New China to control schistosomiasis, which was called “god of plague” by Chairman Mao, the founder of the People's Republic of China. To wipe out “god of plague”, transmission control strategy with emphasis on control of the snail intermediate host was implemented, and mass campaigns were launched with aims to snail control by environmental modification and mollusciciding. At this stage, snail habitats were greatly shrunk, and the number of schistosomiasis cases reduced remarkably [6]. The introduction of praziquantel, a highly effective, low-cost anti-schistosomiasis drug, led to the national schistosomiasis control strategy shifting from transmission control to morbidity control (from mid-1980s to 2003). The governmental commitment to eliminate schistosomiasis and the 10-year implementation of the World Bank Loan Project for Schistosomiasis Control (1992–2001) that provided sufficient funds resulted in great achievements [7, 8]. During this stage, Shanghai municipality, Guangxi Autonomous Region and Fujian, Guangdong and Zhejiang provinces achieved the national criteria for elimination of schistosomiasis [9]. However, the termination of the World Bank Loan Project for Schistosomiasis Control led to a sharp decline in the funds allocated to schistosomiasis control. In addition, many anti-schistosomiasis institutions were incorporated into general centre for disease control and prevention (CDC) systems around the year 2000, and the professional teams in schistosomiasis were greatly weakened. Such a situation resulted in a resurgence of schistosomiasis japonica in China [10, 11]. In 2004, Chinese government made schistosomiasis one of the highest priorities in communicable disease control, and a new integrated strategy with emphasis on the control of infectious sources was developed to control the transmission of *S. japonicum* [12]. The control interventions have been made possible due to the strong governmental will to eliminate the disease by 2020 and adequate investment in public health owing to China’s rapid economic development, and the strategy has been shown high effective [13–15]. By 2012, the prevalence of *S. japonicum* infection in both human and livestock has been decreased to less than 1% in most endemic counties [16]. From the history of schistosomiasis control in China, it is considered that reduced political will and financial support may pose a great threat to the progress of schistosomiasis elimination [17].

Climate change has been proved a driver of the transmission of vector-borne diseases [18, 19], and it has been demonstrated that schistosomiasis, a snail-borne parasitic disease, appears to be affected by the climate change [20, 21]. In China, the geographical distribution of the snail host *O. hupensis* is restricted to areas where the mean January temperature is above 0°C [22]. The rise of temperature may cause snail survival and reproduction in currently non-breeding sites, resulting in the potential transmission of schistosomiasis. It was predicted that the temperature increases by 0.9°C in 2030 and 1.6°C in 2050 in China, which may expand schistosomiasis transmission into currently non-endemic areas in the north, with an additional 783883 km^2^ area [23], and 20.7 million people at risk of infection by 2050 [24]. It is therefore considered that climate change may affect, in a long-term manner, the progress of schistosomiasis elimination in China.

Praziquantel is currently the only drug of choice for the treatment of human schistosomiases. Evidence from Africa and South America has shown that resistance to praziquantel may emerge following long-term, extensive and repeated use [25–27]. In China, praziquantel-based chemotherapy has been implemented to control the morbidity and reduce the prevalence and intensity of *S. japonicum* infection for nearly 4 decades, and the strategy has been proved highly effective [28]. There is therefore a great concern about the development of drug resistance [29]. We evaluated the sensitivity of *S. japonicum* to praziquantel in highly, moderately and lowly endemic regions of China, and no evidence of resistance to praziquantel was detected [30, 31]. However, there are still some cases infected with *S. japonicum* in whom standard treatment regimen fails to clear the infection [32]. In addition, it has been proved that *S. japonicum* subjected to drug pressure may develop resistance to praziquantel [33]. The potential emergence of praziquantel resistance would seriously threaten the elimination of schistosomiasis in China, since praziquantel-based chemotherapy is still critical to the national schistosomiasis control program and no alternatives have been developed. Identification of populations at a high risk of praziquantel resistance is of urgent need.

China is only endemic for *S. japonicum. Biomphalaria straminea*, an intermediate host of *S. mansoni* in South America, was first discovered in southern China in 1981 as an invasive snail species, and the snail habitants appear extensive expansion [34]. With the continuous rise in the detection of schistosomiasis cases caused by *S. manosni* in mainland China, the likelihood of transmission of schistosomiasis mansoni in China receives worldwide attention [35]. The lack of highly effective diagnostics, high mobility of the imported cases infected with *S. mansoni*, and their non-specific syndromes which may easily lead to missed diagnosis and misdiagnosis, and the lack of a sensitive surveillance-response system make it extremely difficult to tackle the risk of the transmission of *S. mansoni* in China, which poses a threat to the progress of schistosomiasis elimination in China [36]. Determination of the compatibility of *B. straminea* present in southern China to *S. mansoni*, and the susceptibility of the cercariae released from *B. straminea* to definitive hosts, should be given high priority.

Although these factors may have a potential impact on the progress towards the elimination of transmission of schistosomiasis in China, we do believe the goal of schistosomiasis elimination can be achieved in China by 2020 with the strong political and financial support from the Chinese Government and enhanced multi-sector collaborations.
